# HPV profiles in Botswana: An analysis of healthy women, cervical intraepithelial neoplasia, and invasive cervical cancer^☆^

**DOI:** 10.1016/j.gore.2025.101971

**Published:** 2025-10-17

**Authors:** Caroline Kernell, Emily MacDuffie, Xiang Lin, Le Gao, Doreen Ramogola-Masire, Surbhi Grover, Erle Robertson

**Affiliations:** a University of Texas at Southwestern Medical Center, Dallas, TX, United States; b Department of Radiation Oncology, Perelman School of Medicine, University of Pennsylvania, Philadelphia, PA, United States; c New Jersey Institute of Technology, Newark, NJ, United States; d University of Botswana, Gaborone, Botswana; e Departments of Otorhinolaryngology-Head and Neck Surgery, and Microbiology, and the Abramson Cancer Center, Perelman School of Medicine at the University of Pennsylvania, Philadelphia, PA, United States

**Keywords:** Human papilloma virus, Cervical cancer, Cervical intraepithelial neoplasia, HIV, Botswana, PathoChip microarray

## Abstract

**Purpose/Objective(s):**

High prevalence of HPV and HIV contribute to the high rate of cervical cancer (CaCx) in Botswana. HPV subtypes in healthy, unvaccinated students (Cohort 1), women with CIN II/III (Cohort 2), and women with invasive CaCx (Cohort 3) were compared.

**Materials/Methods:**

The Ipabalele study in Gaborone, Botswana enrolled patients between 2016–2020. Demographics, clinical characteristics, and HPV cervical swabs were collected. PathoChip quantified prevalence of HPV subtypes.

**Results:**

Overall 414 patients enrolled. Cohort 1: 43; Cohort 2; 212; Cohort 3: 159. Median age was 19, 39, and 46 years. Women living with HIV (WLWH) accounted for 0 %, 76 %, and 72 %. High-risk (HR) HPV prevalence in Cohort 1 increased 34 % to 57 % over 20 months. HPV profiles did not differ by HIV status. The prevalence of all HR HPV subtypes in Cohorts 2 and 3 is increased compared to Cohort 1. The prevalence of HPV HR subtypes except for HPV 53 is increased in Cohort 3 compared to Cohort 2. Among WLWH, Cohort 3 had a higher prevalence of HPV 16, 18, and 34 compared to Cohort 2. Among women without HIV, Cohort 3 had a higher prevalence of HPV 16 and 18 compared to Cohort 2.

**Conclusions:**

Compared to healthy women, HPV subtype representation is higher among women with CIN and CaCx. This suggests the presence of multiple HR HPV strains may impact transformation from pre-cancerous lesions to CaCx, highlighting the importance of CIN detection and primary prevention of HR HPV to decrease the incidence of CaCx in Botswana.

## Highlights

• Widespread HIV and HPV in Sub-Saharan Africa contributes to the growing burden of cervical cancer.
• Compared to healthy students, women with CIN or invasive cervical cancer have a higher prevalence of high-risk (HR) HPV.
• Compared to women with CIN, women with invasive cervical cancer have higher prevalence of HR HPV.
• Cellular HPV viral burden remains unchanged between healthy students, women with CIN, and women with cervical cancer.
• Regardless of viral burden, further acquisition of HR HPV in those with CIN is important in progression to cervical cancer.

## 1 Introduction

Human papilloma virus (HPV) is a sexually transmitted infection that causes cervical cancer (Zur Hausen, 1994, Bosch et al., 1995, Szymonowicz and Chen, 2020). Most cervical cancers are associated with HPV subtypes 16 and 18, however in women with human immunodeficiency virus (HIV) infection, a larger proportion are associated with other high-risk subtypes. Co-infection of HIV with HPV is associated with a 6-fold increase in cervical cancer risk (Stelzle et al., 2021). Because the burden of HIV infection is concentrated in low- and middle-income countries (LMICs), understanding HPV pathogenesis in high-HIV settings is critical to address the global cervical cancer burden. A majority of cervical cancer deaths occur in LMICs in Sub-Saharan Africa (SSA) (Bray et al., 2018). Botswana, a middle-income country of 2.6 million people, has a high cervical cancer incidence, driven by its high HIV infection rate of 20 % (Botswana U, xxxx, xxxx). Since the implementation of free antiretroviral therapy (ART) for citizens in 2002, 84 % of patients living with HIV have access to ART and viral suppression is estimated to be 96 % (Ramogola-Masire et al., 2020). With recent expansion in HPV vaccination and screening programs, there were 57 VIA clinics, 45 cryotherapy clinics, and 35 colposcopy LEEP clinics in Botswana as of 2021 (Luckett et al., 2023). For locally-advanced cervical cancer patients, a gynecological multi-disciplinary team clinic (MDT) was established in 2015 to streamline care and reduce treatment delays for women with gynecological malignancies (Grover et al., 2023, Grover et al., 2017). Despite these recent interventions, cervical cancer remains the leading cause of cancer-related death in Batswana women (Grover et al., 2015).

Studies from SSA examining HPV prevalence in cervical cancer patients have primarily focused on high-risk subtypes and lacked quantitative data beyond presence or absence (Dols et al., 2012, Mpunga et al., 2020, Menon et al., 2016, Okoye et al., 2021). Similarly in Botswana, the prevalence of high-risk subtypes have been described in several different demographic groups. A cross-sectional study reported over 60 % of unvaccinated university-age women carried HPV, with 16 % carrying HPV 16 or 18, and 44 % carrying a non-high risk subtype (Ramatlho et al., 2022). Among patients in Botswana with invasive cervical cancer, forty HPV subtypes have been identified, with the most prominent subtypes being high-risk subtypes HPV 34 and 26 (Grover et al., 2023). These studies have notably been limited in their ability to quantify the presence of individual subtypes, hindering their ability to compare the distribution of viral burden in different populations.

This study recorded the prevalence and quantified the burden of 5 high-risk and 35 low-risk HPV subtypes among three distinct demographic cohorts of women in Botswana to better understand the shifts in HPV pathogenesis that may impact subsequent development of cervical dysplasia and invasive cancer. These three cohorts included healthy, HPV-unvaccinated university-aged women, women diagnosed with precancerous cervical intraepithelial neoplasia (CIN), and women diagnosed with invasive cervical cancer. This study used a novel enhanced HPV subtyping technology to quantitatively compare HPV burden and prevalence across these cohorts of women in Botswana. Describing the landscape of HPV subtype distribution in diverse cohorts with empirical data is critical to guiding efforts at HPV prevention with region-appropriate vaccination initiatives.

## 2 Materials and methods

### 2.1 Cohort enrollment and data collection

The Ipabalele study and enrolled three cohorts of subjects between 2016–2020 in Botswana (Grover et al., 2019). The FHUS Cohort consisted of female healthy university students (FHUS) over 18 years old. Baseline demographic data, urine sample, HIV test, and baseline cervical swab were collected. Three additional cervical swabs were collected at 3–6 months, 9–12 months, and 16–20 months. The CIN II/III Cohort consisted of women with histologically confirmed high grade CIN (CIN2/3). Baseline demographic data and cervical swab were obtained. The CaCx Cohort consisted of women with histologically confirmed invasive cervical cancer (CaCx) receiving curative-intent treatment. Participants were enrolled at Princess Marina Hospital’s women’s gynecological health clinic as well as at Gaborone Private Hospital. Baseline and follow-up patient and disease characteristics, treatment toxicities, blood, and cervical swab were obtained.

### 2.2 PathoChip

The PathoChip is a functional microarray assay with probes able to detect both unique and conserved genomes of numerous organisms. The use of PathoChip was previously documented in studies (Banerjee et al., 2015, Baldwin et al., 2014). Using these probes, we tested for high-risk HPV subtypes 16, 18, 26, 34, and 53; and low-risk subtypes 1, 2, 4, 5, 6b, 7, 9, 10, 32, 41, 48, 49, 50, 54, 60, 61, 63, 88, 90, 92, 96, 101, 103, 108, 109, 112, 116, 121, 126, 128, 129, 131, 132, 134, and 148. HPV 41 was not among the signature probes tested for The CIN II/III Cohort.

### 2.3 Sample storage, preparation, and microarray processing

Cervical swab samples were stored in phosphate-buffered saline solution at −80 °C until processing. The array design and screening procedure for PathoChip has been previously described in multiple studies (Grover et al., 2023, Banerjee et al., 2015, Baldwin et al., 2014, Ramogola-Masire et al., 2022, Raesima et al., 2015, Banerjee et al., 2018, Banerjee et al., 2017, Banerjee et al., 2017).

### 2.4 Microarray data extraction and statistical analysis

Data extraction and analysis after detection of signals using Agilent scanner D was previously described. A description of the analysis pipeline was also previously described (Grover et al., 2023, Banerjee et al., 2015, Baldwin et al., 2014, Ramogola-Masire et al., 2022, Raesima et al., 2015).

### 2.5 Clinical data analysis

Hybridization signal intensity (HSI) and prevalence was determined for each HPV subtype by cohort. Prevalence was determined by tallying samples with signals exceeding the average signal of the human probes (using a cutoff) and expressing it as a percentage of the total sample count. The FHUS Cohort samples from the four timepoints was compared using a Chi-squared test. The CIN II/III Cohort and 3 samples were stratified by HIV status and compared using a Chi-squared test. The distributions of the different HPV subtypes were compared between the three cohorts, severity of cervical dysplasia, and FIGO stage of cervical cancer. All analysis was conducted using R version 4.1.0.

### 2.6 Ethics

This study was approved by the University of Pennsylvania (IRB # 822837), the Human Research Development Council at the Ministry of Health and Wellness in Botswana, and the University of Botswana institutional review boards.

## 3 Results

### 3.1 Demographic and clinical characteristics

A total of 414 patients were included in this study, full demographic characteristics are shown in Table 1. The FHUS Cohort included 43 patients with a median age of 19 (IQR 19–19.5). Women living with HIV (WLWH) accounted for 0 % (n = 0) of the FHUS Cohort. The CIN II/III Cohort included 212 patients with a median age of 39 (IQR 35–45). WLWH accounted for 76 % (n = 161) of the CIN II/III Cohort. The CaCx Cohort included 159 patients with a median age of 46 (IQR 41–53). WLWH accounted for 72 % (n = 115) of the CaCx Cohort.

**Table 1:** Demographic characteristics of Cohorts 1, 2, and 3.

Total	Cohort 1		Cohort 2						Cohort 3					
	All		All		HIV +		HIV −		All		HIV +		HIV −	
	N	Percentage (%)	N	Percentage (%)	N	Percentage (%)	N	Percentage (%)	N	Percentage (%)	N	Percentage (%)	N	Percentage (%)
	43	100.0	212	100.0	161	100.0	51	100.0	159	100.0	115	100.0	44	100.0
Age (y)														
</=20	43	100.0	0	0.0	0	0.0	0	0.0	0	0.0	0	0.0	0	0.0
20–40	0	0.0	64	30.2	49	30.4	15	29.4	34	21.4	28	24.3	6	13.6
40–60	0	0.0	50	23.6	41	25.5	9	17.6	107	67.3	81	70.4	26	59.1
>60	0	0.0	2	0.9	2	1.2	0	0.0	18	11.3	6	5.2	12	27.3
Median	19		39		40		36		46		45		52	
IQR	19–19.5		35–45		36–46		33–45		41–53		41–50		456–62	

Height (cm)														
</=150	1	2.3	2	0.9	2	1.2	0	0.0	9	5.7	8	7.0	1	2.3
150–160	14	32.6	53	25.0	43	26.7	10	19.6	55	34.6	41	35.7	14	31.8
160–170	22	51.2	54	25.5	41	25.5	13	25.5	67	42.1	47	40.9	20	45.5
>170	6	14.0	7	3.3	6	3.7	1	2.0	14	8.8	8	7.0	6	13.6
Median	161.4		161.0		161.0		161.0		161.5		161.0		163.0	
IQR	156.4–166.2		157–165		157–165		158.8–165		156.2–167		155.9–164.8		158.5–167	

Weight (kg)														
</=50	18	41.9	12	5.7	11	6.8	1	2.0	16	10.1	15	13.0	1	2.3
50–60	12	27.9	31	14.6	25	15.5	6	11.8	32	20.1	27	23.5	5	11.4
60–70	8	18.6	34	16.0	31	19.3	3	5.9	30	18.9	25	21.7	5	11.4
>70	5	11.6	39	18.4	25	15.5	14	27.5	67	42.1	38	33.0	29	65.9
Median	54.9		64.0		62.1		71.9		68.9		64.9		79.3	
IQR	46.5–64		55.9–73.1		54.2–71.3		60–78.4		56.4–79.8		54.6–74.4		68.3–88.9	

BMI														
Underweight (<18.5)	9	20.9	6	2.8	6	3.7	0	0.0	10	6.3	10	8.7	0	0.0
Healthy weight (18.5–24.9)	27	62.8	53	25.0	44	27.3	9	17.6	55	34.6	46	40.0	9	20.5
Overweight (25.0–29.9)	4	9.3	37	17.5	29	18.0	8	15.7	36	22.6	27	23.5	9	20.5
Obese (>/=30)	3	7.0	20	9.4	13	8.1	7	13.7	39	24.5	18	15.7	21	47.7
Median	20.9		25.0		24.5		28.0		25.7		24.7		31.1	
IQR	18.7–23.8		21.1–27.9		20.9–27.0		23.6–30.9		22.4–31.1		21.5–29.2		25.7–34.0	

Age of Sexual Debut (y)														
</=18	17	39.5	53	25.0	41	25.5	12	23.5	84	52.8	60	52.2	24	54.5
>18	11	25.6	49	23.1	40	24.8	9	17.6	54	34.0	37	32.2	17	38.6
No sex	15	34.9	110	51.9	80	49.7	30	58.8	21	13.2	18	15.7	3	6.8
Median	18		18		18		18		18		18		18	
IQR	18–19		17–20		17–20		18–20		17–20		17–20		17–20	

Number of Sexual Partners														
1	16	37.2	3	1.4	3	1.9	0	0.0	17	10.7	7	6.1	10	22.7
>1	12	27.9	99	46.7	78	48.4	21	41.2	111	69.8	81	70.4	30	68.2
0	15	34.9	110	51.9	80	49.7	30	58.8	21	13.2	18	15.7	3	6.8
Median	1		5		5		3		3		3		3	
IQR	1–2		3–7		3–6		3–7		2–5		2–4		2–5	

Smoking Status														
Yes	6	14.0	9	4.2	5	3.1	4	7.8	3	1.9	2	1.7	1	2.3
No	37	86.0	106	50.0	86	53.4	20	39.2	156	98.1	113	98.3	43	97.7

### 3.2 High-Risk HPV prevalence and burden

**Fig. 1:**
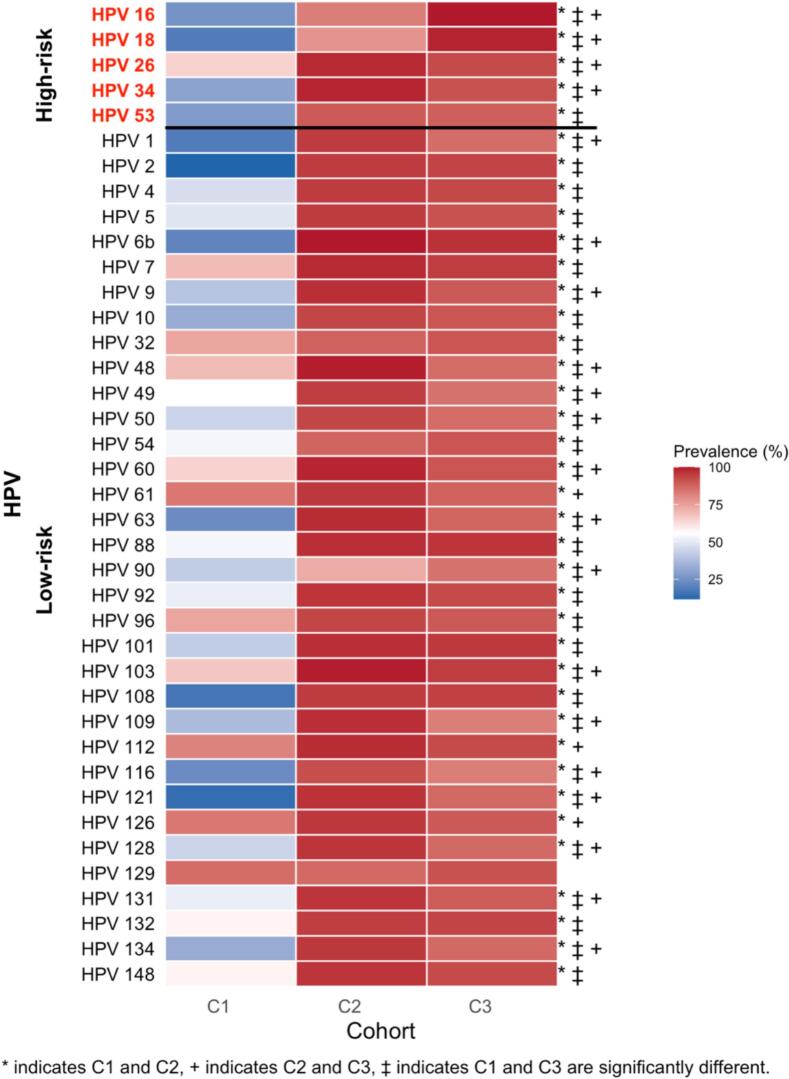
Prevalence of HPV subtypes in Cohorts 1, 2, and 3.

**Fig. 2:**
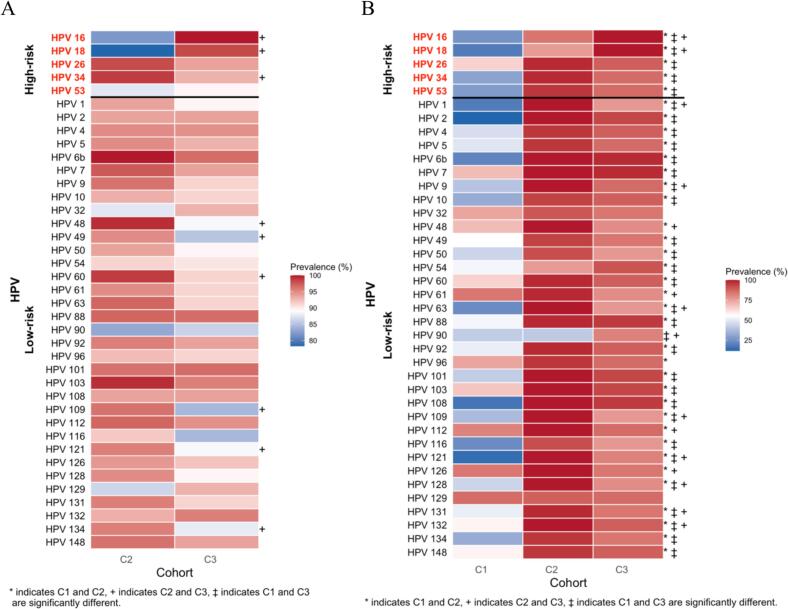
Prevalence of HPV subtypes in Cohorts 1, 2, and 3 by HIV status. 2A: HPV subtypes in WLWH, 2B: HPV subtypes in women without HIV.

**Fig. 3:**
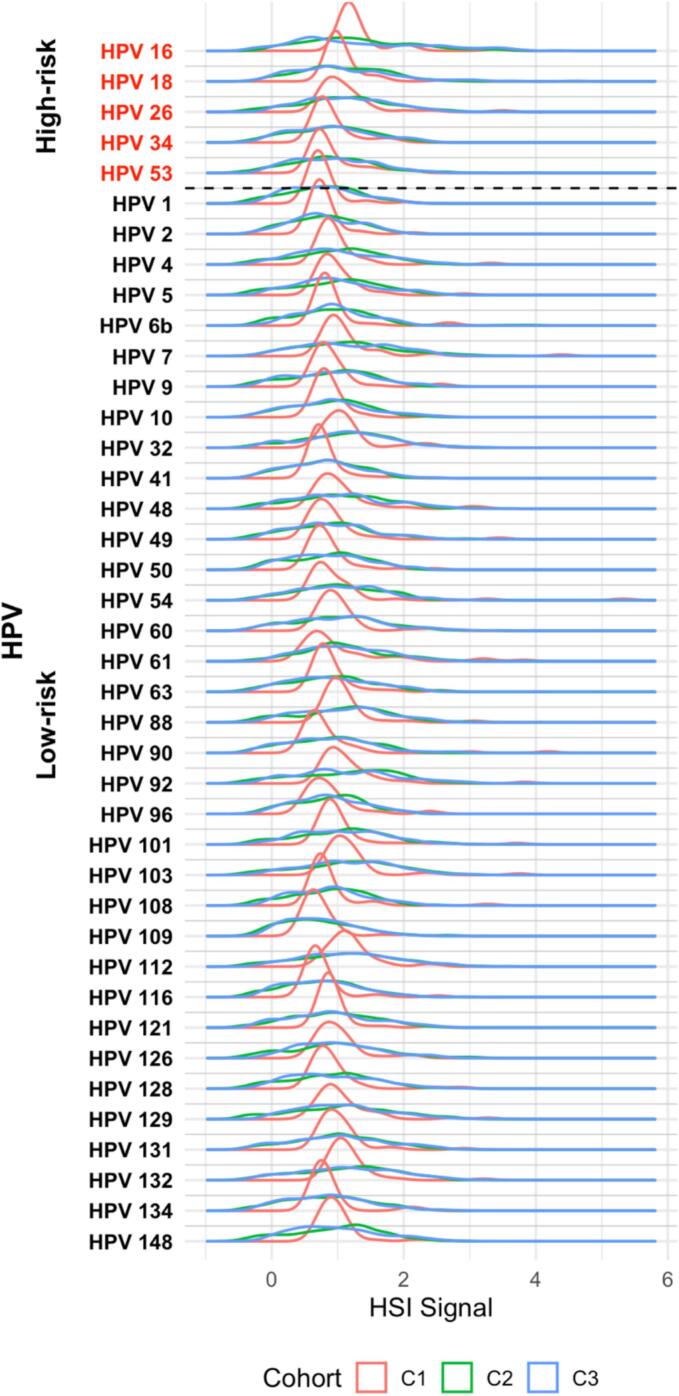
HSI of HPV subtypes in Cohorts 1, 2, and 3.

**Fig. 4:**
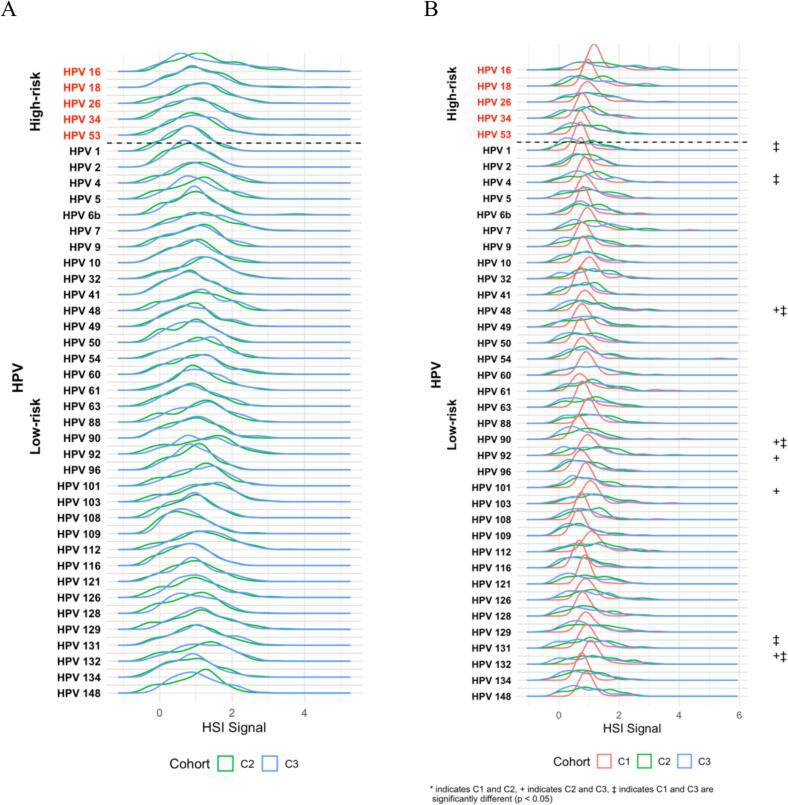
HSI of HPV subtypes in Cohorts 1, 2, and 3 by HIV status. 4A: HPV subtypes in WLWH, 4B: HPV subtypes in women without HIV.

All five high-risk subtypes tested (16, 18, 26, 34, 53) were detected across the three cohorts. The prevalence of these subtypes rose markedly from healthy university students to women with CIN and then to those with invasive cervical cancer (Fig. 1). In the FHUS Cohort, baseline prevalence of HPV 16 and 18 was low (26 % and 19 %), with HPV 26 being the highest (65 %). Among the high-risk subtypes tested, all showed a statistically significant increase in prevalence from baseline to 16–20 months (p < 0.05), with HPV 26 remaining the most prevalent (88 %) (Supplement Table 1). In contrast to the FHUS Cohort, in the CIN II/III Cohort prevalence of HPV 16, 18, 26, 34, and 53 ranged from 78 %-99 %, and in the CaCx Cohort nearly all women carried HPV 16 (100 %) and HPV 18 (98 %) (Supplement Tables 2-3). Compared with the CIN II/III Cohort, the CaCx Cohort showed significantly higher prevalence of HR HPV subtypes HPV 16 and 18 (p < 0.05), with the CIN II/III Cohort having a higher prevalence of other HR HPV 26 and 34 (p < 0.05) (Fig. 1). When stratified by HIV status, WLWH in the CaCx Cohort had higher prevalence of HPV 16 and 18 than WLWH in the CIN II/III Cohort (p > 0.05), and HIV-negative women in the CaCx Cohort had a higher prevalence of HPV 16 and 18 than those in the CIN II/III Cohort (p < 0.05) (Fig. 2A and B). HIV status among the cohorts themselves by HPV subtype are detailed in Supplement Tables 2-3. Hybridization signal intensity (HSI), a surrogate of viral burden, did not differ significantly among cohorts (Fig. 3) or by HIV status (Fig. 4A and B).

### 3.3 Low-Risk HPV prevalence and burden

All thirty-five low-risk HPV subtypes tested were detected across the three cohorts. The prevalence of these subtypes was highest in women with CIN and remained high in those with invasive cervical cancer (Fig. 1). In the FHUS Cohort, the most common LR HPV subtypes at baseline were HPV 61 and 126 (84 % each), followed by a broad range of other low-risk subtypes, many of which increased significantly in prevalence at 16–20 months (p < 0.05) (Supplement Table 1). Between baseline and 16–20 months, most low-risk subtypes showed a new increase in prevalence, though a few (HPV 60, 103, 112, 126, 129, and 148) decreased. In contrast to the FHUS Cohort, the CIN II/III Cohort prevalence of most low-risk subtypes exceeded 85 %, with HPV 6b detected in 100 % of patients and only HPV 90 below 75 % (Supplement Table 2). The CaCx Cohort remained consistent with the CIN II/III Cohort, with 97 % of patients having HPV 6b infection (Supplement Table 3). Compared with the CIN II/III Cohort, the CaCx Cohort showed a significantly higher prevalence of HPV 90 (p < 0.05), with most other LR HPV types being significantly more prevalent in the CIN II/III Cohort (p < 0.05). (Fig. 1). When stratified by HIV status, WLWH in the CIN II/III Cohort had a significantly higher prevalence of low-risk subtypes HPV 48, 49, 60, 109, 121, 134 when compared to the CaCx Cohort (p < 0.05), while HIV-negative women in the CIN II/III Cohort had higher prevalence of numerous low-risk subtypes compared to both Cohorts 1 and 3 (Fig. 2A and B). HIV status among the cohorts themselves by HPV subtype are detailed in Supplement Tables 2-3. HSI did not differ significantly among cohorts (Fig. 3) or by HIV status (Fig. 4A and B).

## 4 Discussion

This study compared the distribution, prevalence, and burden of HPV subtypes between healthy university students, patients with CIN 2/3, and patients with invasive cervical cancer undergoing curative treatment in Botswana.

### 4.1 HPV subtype distribution across the cervical cancer spectrum

Our data show a rise in prevalence of HR HPV from healthy university students to women with CIN 2/3 and then to those with cervical cancer, while viral burden (HSI) remained stable across cohorts and HIV status. This finding reinforces the concept that persistent infection with oncogenic HPV subtypes, rather than absolute viral load, drives progression to cervical cancer. This finding may suggest that ART, which has a high level of adherence in Botswana, surpassing 95 % since the inception of a widespread HIV testing and ART program implemented by the government in Botswana in 2002, (Development CfGCfG, 2016) may be playing a protective role in controlling HPV viral load, even though HIV infection remains a recognized risk factor for cervical cancer (Stelzle et al., 2021).

### 4.2 The FHUS Cohort: University students and high-risk HPV acquisition

The HPV subtype prevalence seen in the FHUS Cohort builds on the results of previous studies of university students in Botswana. A 2022 study investigated for the first time the prevalence of HPV in unvaccinated female university students, finding that 60 % of students were infected with HPV (Ramatlho et al., 2022). Within this population, HPV 26 was the most prevalent, paired with gradual rises in HPV 16 and 18. A mid-study dip followed later by an increase in HPV prevalence suggests both spontaneous clearance and repeated acquisition of infection, as younger women are more likely to clear HPV infection compared to older women (Erickson et al., 2013). These observations further support the continued need for HPV vaccination among this population. Although Botswana initiated the quadrivalent HPV vaccine program for school-going girls ages 9–13 in 2015, uptake was only 47 % as of 2019 (Department SR, 2016). Thus, HPV infection and re-infection remains an issue of high concern for university-age students in Botswana.

### 4.3 Comparison of CIN and invasive cancer

Women with CIN were infected with a broad spectrum of both HR and LR HPV subtypes, whereas those with invasive cervical cancer demonstrated dominance of HPV 16 and 18. Low-risk subtypes were more prevalent in CIN and less prevalent in invasive disease, supporting the view that malignant transformation is characterized by selective persistence of high-risk HPV subtypes rather than overall diversity of infection. However, there is extreme genetic heterogeneity in the distribution of LR HPV subtypes among the 3 cohorts, suggesting a need for further investigation on the differential impact of LR HPV combinations and their influence on carcinogenesis. These trends were similar in women with and without HIV, indicating that, although WLWH are 6 times more likely to develop cervical cancer, they do not require a lower viral burden of HPV to develop CIN compared to their HIV negative counterparts. This point once again emphasizes the need for HPV prevention and CIN detection, because regardless of HIV status, women are at equal risk for development of these lesions.

### 4.4 Implications for vaccination and screening

Botswana’s high ART coverage and the success of its HPV vaccination program represent major public health advances, yet a substantial population still remains at risk for HPV exposure. Girls vaccinated in 2015 are now in their mid-20 s, leaving a large population of unvaccinated young adults still vulnerable to HPV and cervical cancer. The subtypes in the FHUS Cohort also suggest the need to shift to the nonavalent vaccine, which covers HPV 6, 11, 16, 18, 31, 33, 45, 52, and 58. Although this study did not examine all subtypes the vaccine covers, the rising prevalence of non-16/18 HR HPV in young women indicates other subtypes, such as HPV 34, are contributing to disease. Importantly, this study shows that acquisition of high-risk HPV in women with CIN, regardless of HIV status or viral burden, is key in progressing to cancer. This supports early interventions, including vaccinations. Strengthening screening programs and ensuring a high uptake for vaccination will be essential to further reduce the cervical cancer burden in Botswana.

### 4.5 Strengths and limitations

A strength of the study is its use of PathoChip, which detects extremely low viral loads and enables detailed analysis of lesser-known low-risk subtypes. This technology allowed us to capture a broader and more nuanced picture of HPV diversity than is typically possible with standard assays. Additional strengths include the prospective design of the university cohort and the inclusion of both HIV positive and negative patients.

Limitations of this study include potential recall bias with self-reported sexual history, incomplete HPV subtype coverage, and lack of timing data between HIV infection and cervical disease progression. Additionally, since younger women are more likely to clear HPV infections, age differences may possibly account for lower HPV prevalence in the university cohort.

## 5 Conclusions

This study analyzed a wide array of HPV subtypes and prevalence over three different cohorts in Botswana. HR HPV prevalence in university students increased significantly over 20 months. We have also demonstrated a dramatic increase in HPV burden between young and middle-aged women, with women with cervical cancer having the highest prevalence of HR HPV 16 and 18. Lastly, this study also shows the extreme genetic variability among HPV subtypes in this population, and further supports the need for continued and expanded vaccination and added screening protocols for Botswana.

## CRediT authorship contribution statement

**Caroline Kernell:** Writing – review & editing, Writing – original draft, Visualization. **Emily MacDuffie:** Writing – review & editing. **Xiang Lin:** Methodology, Investigation, Formal analysis, Data curation, Conceptualization. **Le Gao:** Methodology, Investigation, Formal analysis, Data curation. **Doreen Ramogola-Masire:** Writing – review & editing. **Surbhi Grover:** Writing – review & editing, Supervision, Funding acquisition. **Erle Robertson:** Writing – review & editing, Supervision.

## Declaration of competing interest

The authors declare the following financial interests/personal relationships which may be considered as potential competing interests: SG reports consulting fees for Lumonus, Harbinger Health, and Sustainable Dialogue for Peaceful Uses (SDPU) for serving as a scientific advisor. All other authors report no conflict of interest.
