# Supplementary material for: HPV profiles in Botswana: An analysis of healthy women, cervical intraepithelial neoplasia, and invasive cervical cancer
Source: Gynecol Oncol Rep. 2025 Oct 17;62:101971. doi: 10.1016/j.gore.2025.101971 (PMC12590286; doi:10.1016/j.gore.2025.101971)

Supplement Table 1: HR and LR HPV subtype prevalence in Cohort 1 over time.


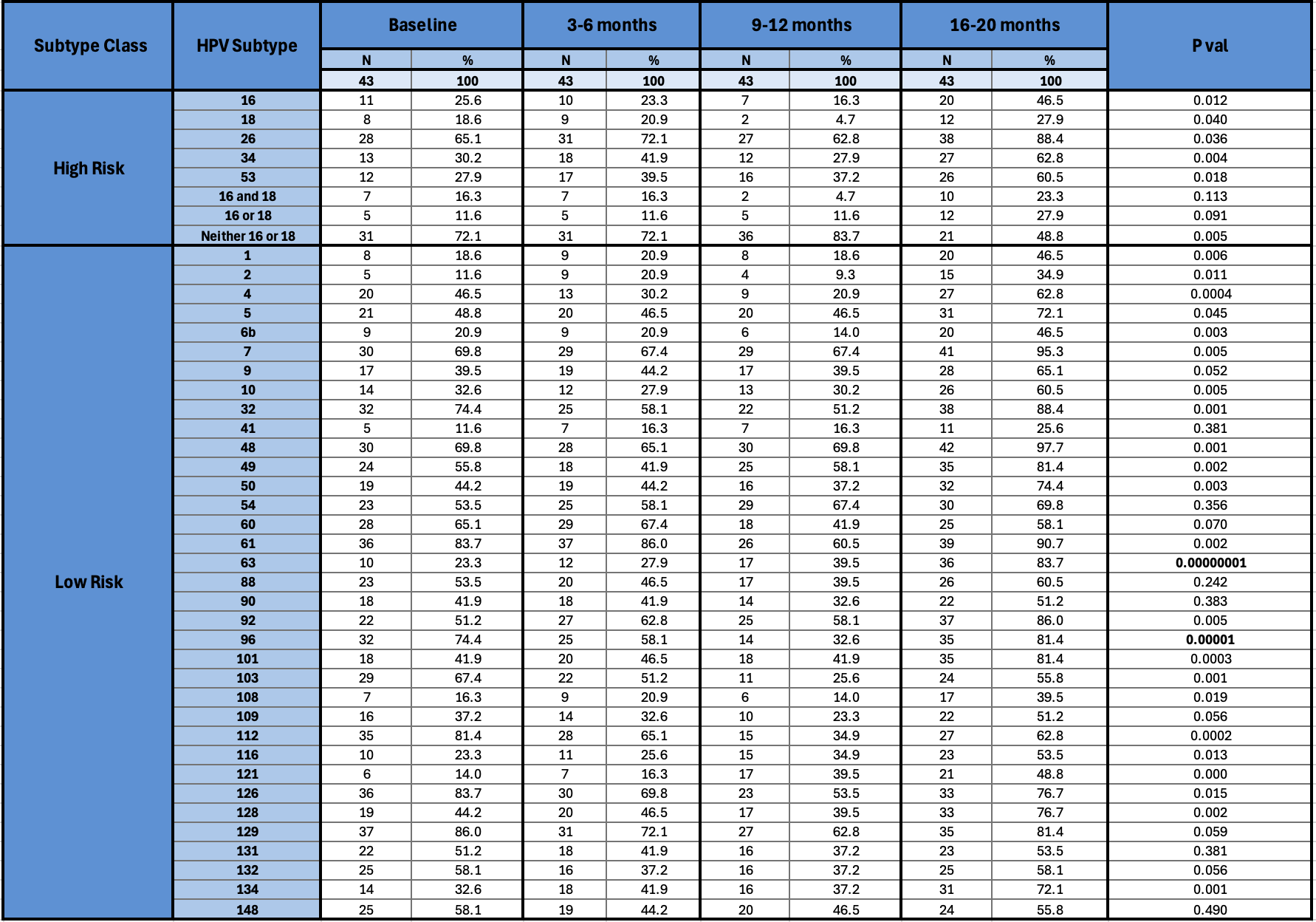


Supplement Table 2: HR and LR HPV subtype prevalence in Cohort 2 by HIV status.


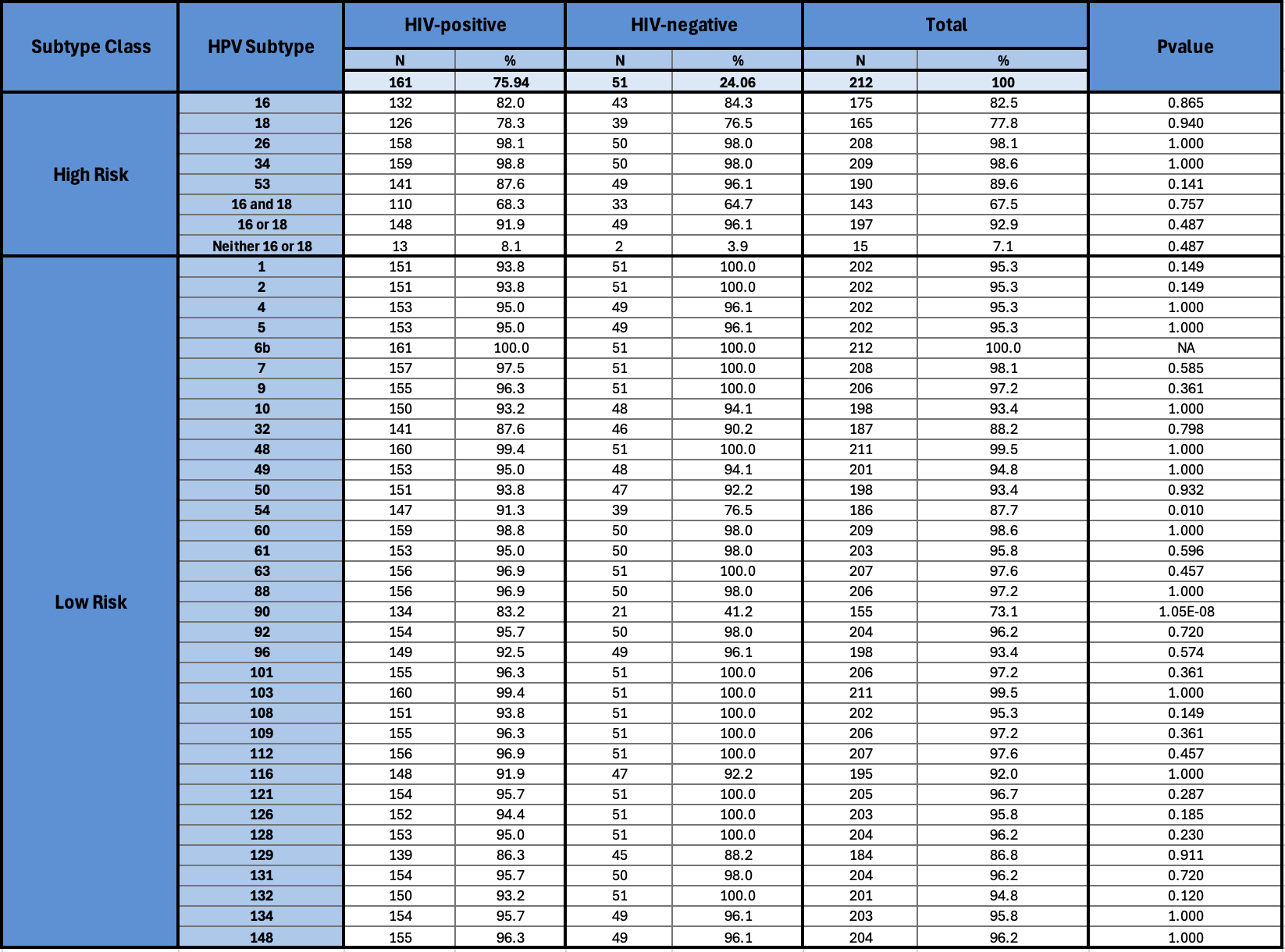


Supplement Table 3: HR and LR HPV subtype prevalence in Cohort 3 by HIV status.


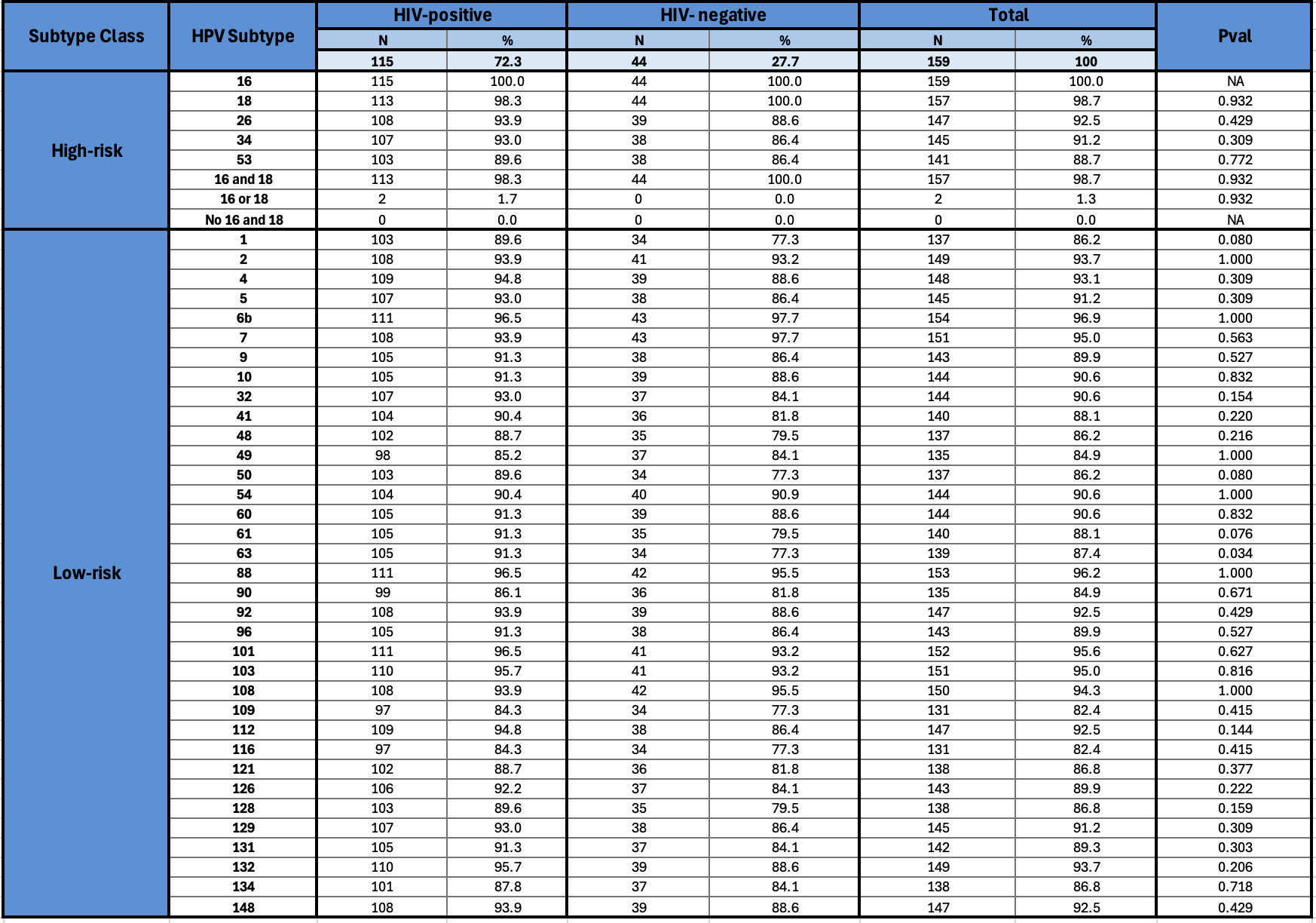


Supplement Table 4: Hybridization signal intensity of high- and low-risk HPV subtypes in Cohort 1


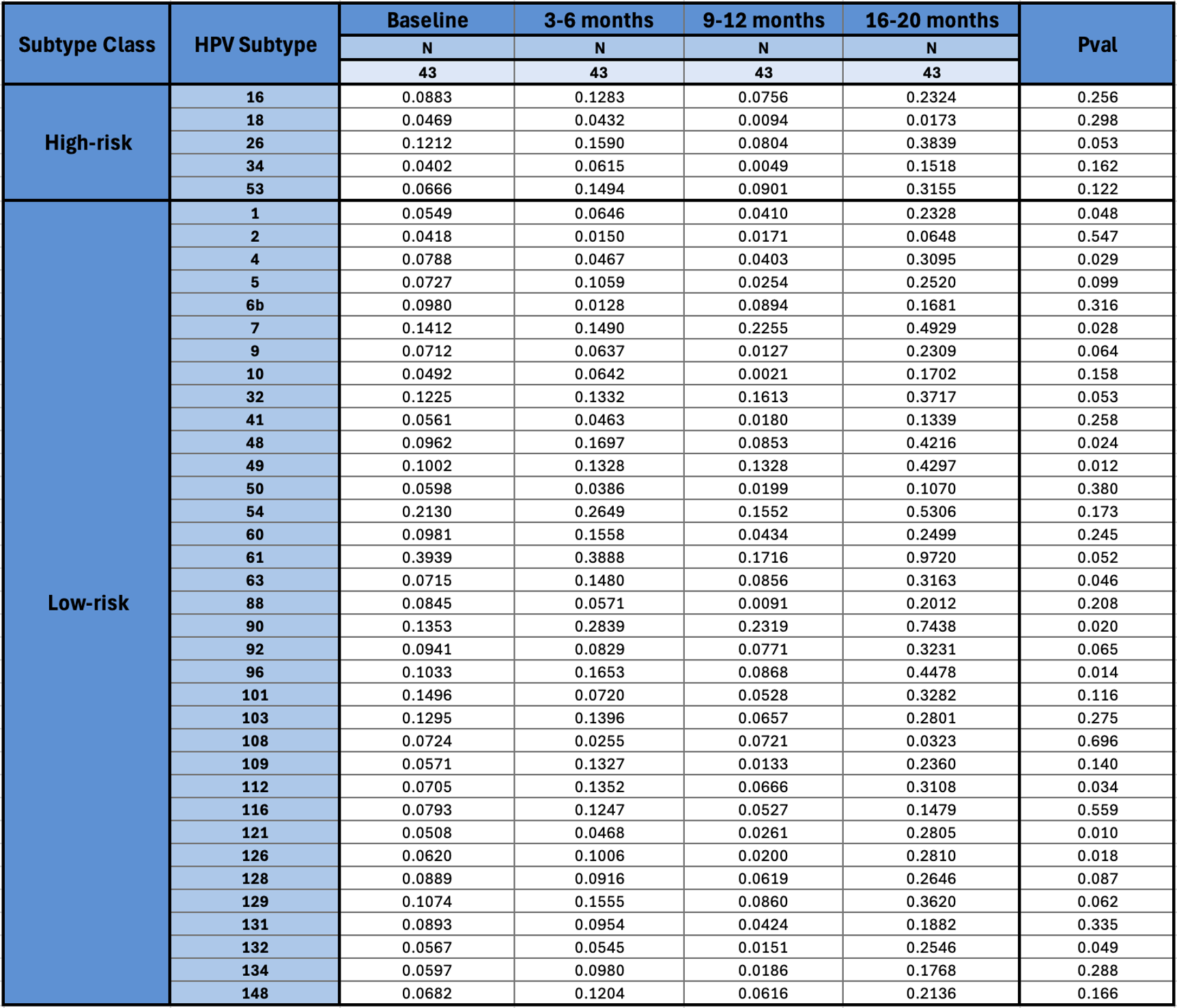


Supplement Table 5: Hybridization signal intensity of high- and low-risk HPV subtypes in Cohort 2 by HIV status


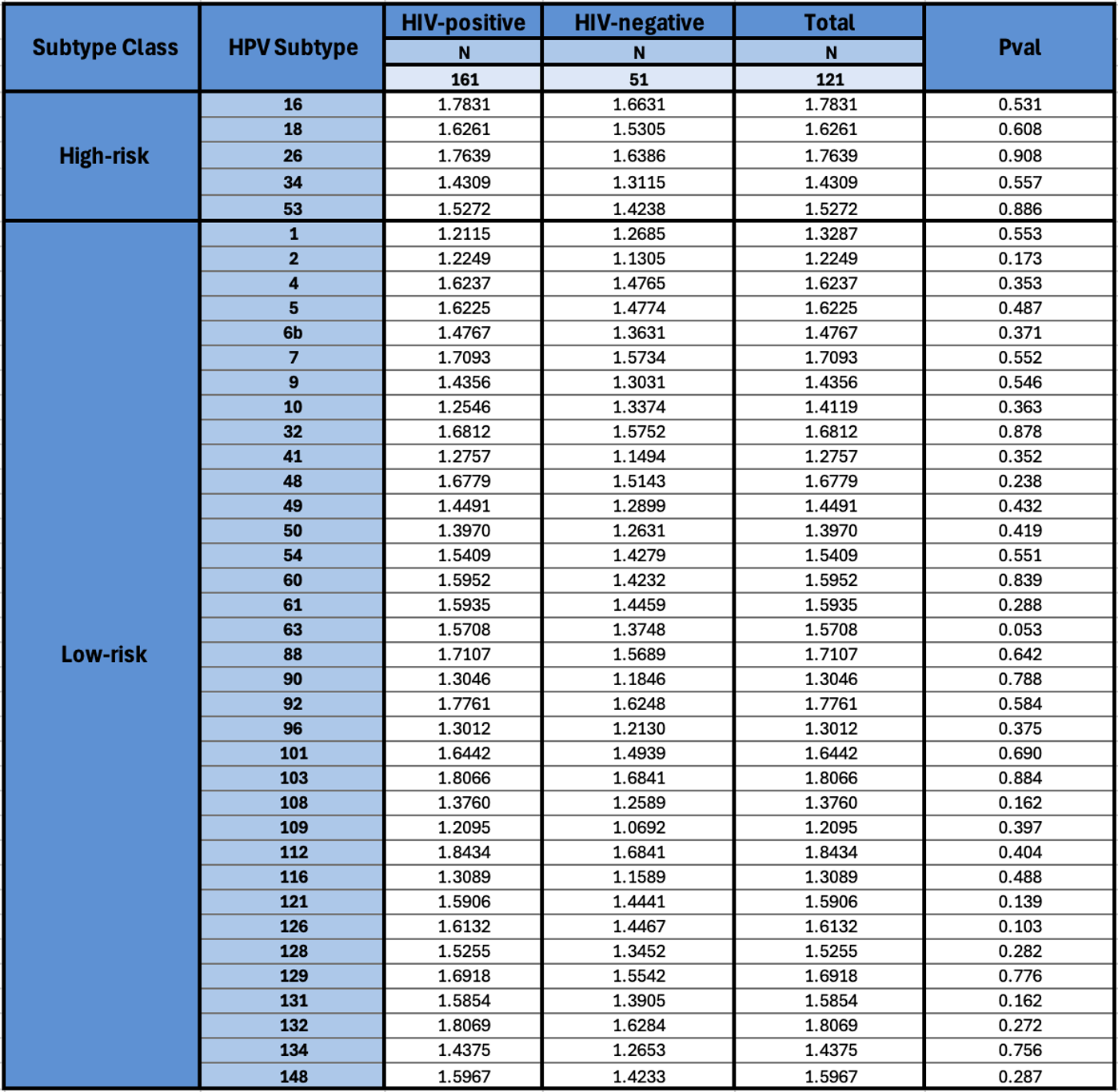


Supplement Table 6: Hybridization signal intensity of high- and low-risk HPV subtypes in Cohort 3 by HIV status


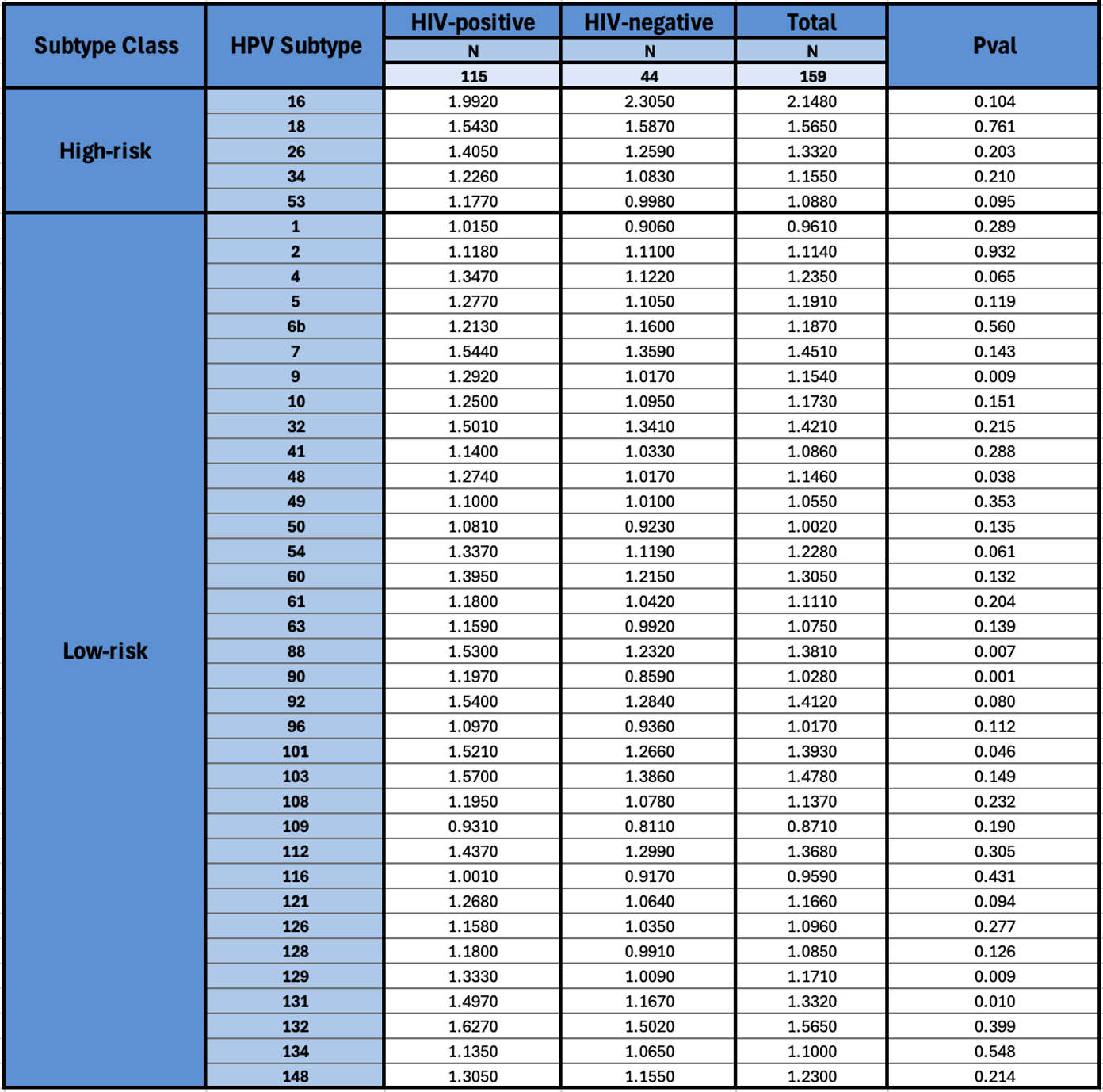

Supplement: Supplementary Data 1 [file mmc1.doc]
